# Mapping Bumblebee Community Assemblages and Their Associated Drivers in Yunnan, China

**DOI:** 10.3390/biology14091222

**Published:** 2025-09-09

**Authors:** Huanhuan Chen, Muhammad Naeem, Licun Meng, Nawaz Haider Bashir, Maryam Riasat, Zichao Liu, Canping Pan

**Affiliations:** 1 College of Biological Resource and Food Engineering, Qujing Normal University, Qujing 655011, China; chhuanhuan@163.com (H.C.);; 2 State Key Laboratory of Resource Insects, Key Laboratory of Insect-Pollinator Biology of Ministry of Agriculture and Rural Affairs, Institute of Apicultural Research, Chinese Academy of Agricultural Sciences, Beijing 100193, China; 3 Department of Zoology, Faculty of Engineering and Applied Sciences, Riphah International University, Faisalabad Campus, Faisalabad 38000, Pakistan; 4 College of Agriculture & Life Sciences, Kunming University, Kunming 650214, China; 5 Department of Applied Chemistry, College of Science, China Agricultural University, Beijing 100193, China

**Keywords:** bioclimatic factors, biogeographic zones, forest, GIS, remote sensing

## Abstract

Bumblebees are among the most important wild pollinators; however, their populations are declining worldwide due to factors such as climate change, habitat loss, and pesticide use. For their conservation, it is important to understand the community structure at the local scale and the drivers responsible for their assemblages. However, little is known about bumblebee community assemblages and their drivers in Yunnan Province, China. In this study, we mapped bumblebee community assemblages across 125 counties in Yunnan Province using field-collected and published data. We also quantified the climatic and land use/land cover (LULC) drivers shaping these assemblages. The climatic habitat suitability for 21 bumblebee species was assessed at the county level across Yunnan using species distribution modeling. The biogeographic zones (groups of counties) were identified using Ward’s agglomerative cluster analysis, and the impacts of 12 bioclimatic and LULC drivers on the zonation pattern were assessed using Canonical Correspondence Analysis (CCA). Results indicated that more than 70% of bumblebee species showed their highest environmental suitability in the northern region of Yunnan. Among climatic factors, temperature-related bioclimatic variables were identified as dominant drivers influencing the spatial distribution of 15 out of 21 bumblebee species within the counties of Yunnan. In contrast, five species, *B. grahami*, *B. impetuosus*, *B. lepidus*, *B. picipies*, and *B. securus*, showed the highest contribution from precipitation-related factors. Six biogeographic zones (I, II, III, IV, V, and VI) were identified using Ward’s agglomerative cluster analysis. All 12 drivers were found to play critical roles in shaping the community assemblages of bumblebee species. This study provides essential insights for devising targeted conservation strategies at a local scale to maintain bumblebee populations in Yunnan.

## Simple Summary

Bumblebees are vital pollinators that help secure food production through their pollination services. However, populations of these wild pollinators are threatened by serious declines worldwide. Therefore, their conservation is important, and to develop effective strategies, it is necessary to understand the community assemblages of these pollinators in specific regions. Unfortunately, little is known about the distribution, community structure, and the environmental factors influencing bumblebee species in Yunnan, China. In the present study, community assemblages of 21 species were assessed across 125 counties in Yunnan Province, China. We identified groups of counties that share similar bumblebee communities and examined how climate and land use shape these patterns. We found that most species were concentrated in the northern parts of Yunnan, where cooler temperatures provide favorable conditions. Our analysis identified six distinct zones across the province, each with different community structures shaped by a combination of environmental factors. These findings highlight the regions most critical for bumblebee survival and offer valuable guidance for protecting their habitats.

## 1. Introduction

Bumblebees are wild pollinators that play an important role in ecosystem stability and food security. They contribute significantly to the pollination of wild plants and agricultural crops, making them essential for agricultural productivity and overall ecosystem health [1,2]. However, their populations are increasingly threatened by various factors, including climate change and alterations in land use/land cover (LULC) structure and composition [2,3,4,5,6]. These threats to bumblebee species underscore the need to implement effective conservation strategies. However, for the successful design and implementation of such strategies, it is crucial to study community structures, species assemblages, and the key drivers influencing these assemblages at a local scale.

Community assemblage refers to the co-occurrence of interacting groups of species within a specific area [7]. Mapping, which is the graphical or spatial representation of these groups of interacting species (assemblages) within a particular region, can help in studying the patterns of assemblages [8]. It can also help us understand the ecological processes occurring at the local scale [9].

Multiple drivers, biogeographic, climatic, biotic, and land use/land cover (LULC), may be responsible for shaping community assemblages [10,11,12,13,14]. For similar species, the contribution of responsible drivers to their assemblages may vary from region to region [14]. For example, some studies have shown the impact of LULC on bumblebee community assemblages [15,16]. A recent study highlighted the contribution of bioclimatic, soil, and vegetation factors to community assemblages [14]. However, the impact of both climate and LULC drivers on bumblebee species remains unknown, particularly in the Yunnan region.

Yunnan Province, covering 4.1% of China’s total area, is renowned for its exceptional biodiversity. It is home to more than 51% of China’s total flora and fauna species, making it a biodiversity hotspot [17]. Despite its rich biodiversity, much of the insect fauna in Yunnan remains understudied, particularly bumblebees, which are key pollinators. Many aspects of their spatial distribution and community structure have yet to be thoroughly explored [18,19]. The current study aims to map the spatial distribution patterns of bumblebee species assemblages across the counties of Yunnan Province, China, and to examine the relative contribution of bioclimatic and land use/land cover (LULC) drivers to these patterns. We hypothesize that both climatic and LULC factors significantly shape bumblebee community assemblages in Yunnan Province. Specifically, we expect that climatic variables will act as the dominant drivers at broader spatial scales, whereas LULC factors will contribute more strongly to local variations in assemblage patterns.

## 2. Materials and Methods

### 2.1. Study Site and Data Collection

**Figure 1:**
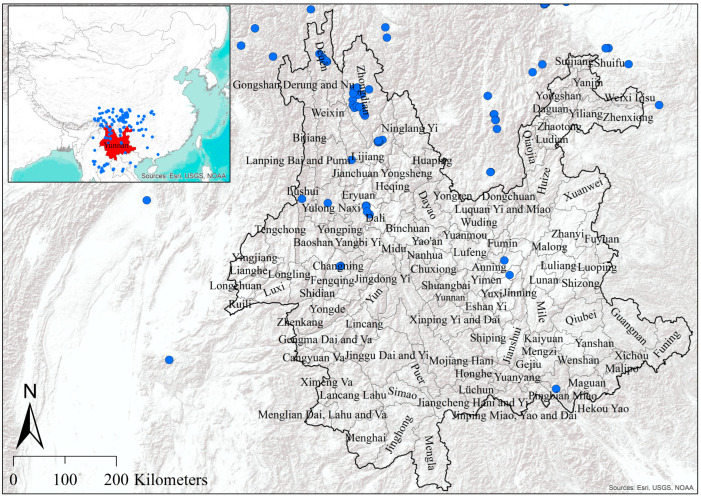
Shaded relief map of administrative boundary of Yunnan Province with 125 counties, China. Blue dots are the collection sites of 21 bumblebee species.

Yunnan province with 125 counties, located in the southwestern part of China, spans between 21°09′–29°15′ N and 97°32′–106°12′ E (Figure 1). Yunnan’s location, at the intersection of three major global biodiversity hotspots, the Indo-Burma region, the mountains of Southwestern China, and the Eastern Himalayas, contributes to its high species richness [20].

To assess the contribution of bioclimatic and LULC drivers in the spatial pattern of bumblebee species within these 125 counties, a dataset of 21 bumblebee species within Yunnan and its surroundings were taken, which includes a total of 1262 collection sites (GBIF Occurrence Download. Available online: https://doi.org/10.15468/dl.xa76us, accessed on 20 December 2024), and some additional records were sourced from the Qujing Normal University collection. To minimize spatial autocorrelation in the data, a spatial rarefaction was applied, and only those records for each species with a distance greater than 10 km^2^ were retained. Duplicate collections were also removed, resulting in a total of 299 records across 21 species (Figure 1).

Bioclimatic data were obtained from www.wordclim.org (accessed on 2 January 2025) for the current time periods. A total of 19 bioclimatic variables were initially considered. Based on Pearson correlation analysis, highly collinear variables were removed, and eight non-correlated variables (r < 0.8) were retained: annual mean temperature (bio1), mean diurnal range (bio2), isothermality (bio3), temperature seasonality (bio4), annual precipitation (bio12), precipitation of the driest month (bio14), precipitation seasonality (bio15), and precipitation of the warmest quarter (bio18). This threshold for removing highly correlated variables is widely used to minimize redundancy, and the filtering process is consistent with standard practices in species distribution modeling [21]. The spatial resolution of these variables was approximately 1 km^2^. ArcGIS v 10.0 (Esri, Redlands, CA, USA) was used to assess the Pearson correlation coefficients between these variables.

Land cover (LULC) data were obtained from the MCD12C1.061 MODIS Land Cover Type Yearly Global product in Google Earth Engine for the year 2024. The Majority_Land_Cover_Type_1 band was used, which provides 17 land cover classes. These 17 classes were reclassified into four categories: vegetation (forests, croplands, savannas, shrublands, and grasslands), urban, barren, and water. This reclassification simplified the interpretation and ensured consistency in our subsequent canonical correspondence analysis (CCA).

### 2.2. Habitat Suitability Assessment Using Species Distribution Modeling and Other Statistical Analysis

The habitat suitability ranges of each bumblebee species were assessed using bioclimatic and LULC variables through the maximum entropy species distribution modeling approach in MaxEnt software (v3.4.4) with one replicate per species [22]. Models with single replicates do not allow direct estimation of uncertainty across replicates (e.g., standard errors or confidence intervals); however, model reliability was instead assessed using the AUC (area under the curve) of the ROC (receiver operating characteristic) statistic [22].

The average habitat suitability ranges of each bumblebee species were calculated for each county. For taking the average of habitat suitability, all the habitat suitability values of all pixels were summed up and then divided with the total number of pixels present within each county. A matrix of species vs. counties (sites) was developed where each row represents a particular county (site) and each column represents a particular species.

Variable importance was assessed using percent contribution, permutation importance, and jackknife tests of regularized training gain from MaxEnt. Response curves were also examined to confirm plausible ecological relationships. A predictor was considered dominant for a species when it ranked highest in permutation importance and was supported either by percent contribution or by the largest reduction in gain when omitted in the jackknife [23,24].

Principal component analysis (PCA) was performed on the bioclimatic variables as an exploratory tool to interpret environmental variation and to visualize the structure of environmental gradients in the study region [25]. For the assemblage and patterns of bumblebees within Yunnan, 125 counties were clustered using Ward’s hierarchical algorithm with Euclidean distance. The number of clusters (k) was determined by evaluating average silhouette width, Calinski–Harabasz index, and Davies–Bouldin index across k = 2–10 [26,27,28].

Potential species richness (based on habitat suitability values) was calculated per county level, and finally, Canonical Correspondence Analysis (CCA) was performed to see the individual variable influence in the clustering or spatial pattern of bumblebee species within Yunnan province, China. Here, CCA was conducted using habitat suitability values of 21 species as community input. Although CCA is conventionally applied to abundance data, we applied it to suitability values because empirical abundance data were incomplete. This approach has already been adopted in other macroecological studies [29]. The significance of the axes in the CCA was tested using permutation tests with 999 iterations. All analyses were carried out using Python 3.12.4. Data manipulation and numerical computations were performed using Pandas and NumPy.

## 3. Results

### 3.1. Habitat Suitability with the Contribution of Bioclimatic and LULC Drivers to Spatial Distribution

**Figure 2:**
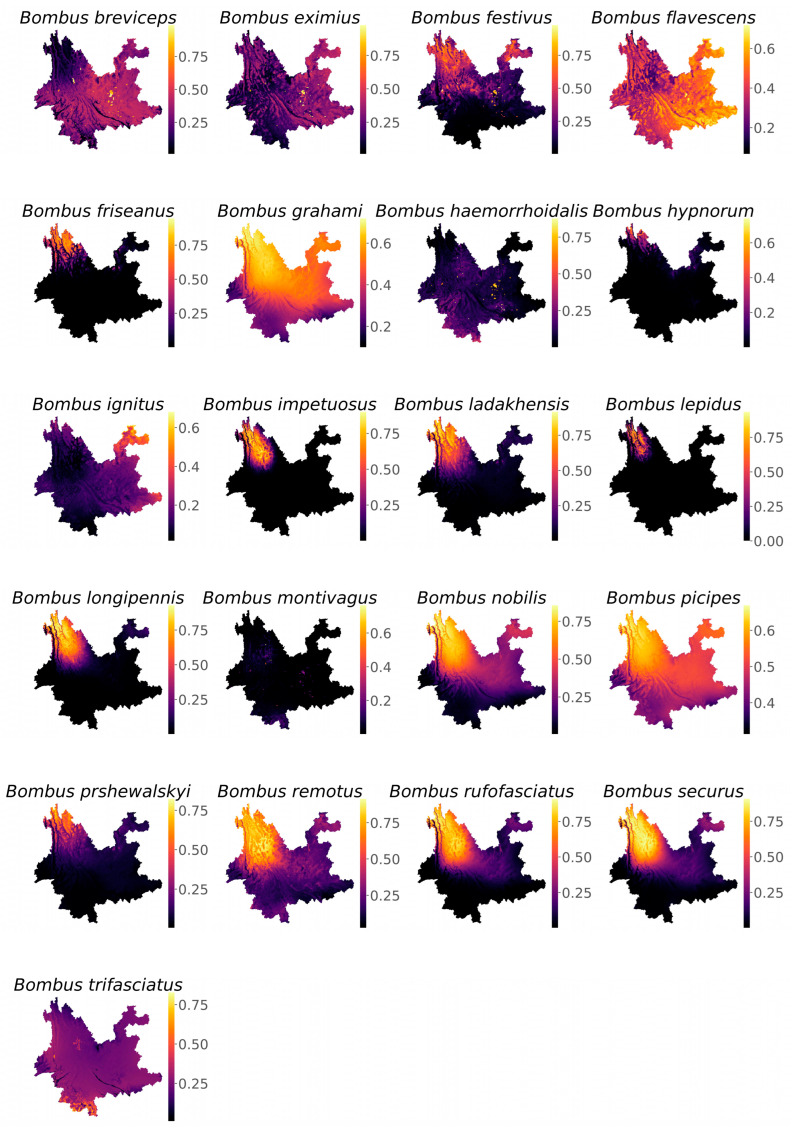
Spatial distribution of habitat suitability for 21 bumblebee species in Yunnan Province, China. Purple/black colors represent the lowest probability values of habitat suitability, whereas pale yellow indicates the highest probability values.

The species distribution models used for habitat suitability analysis by MaxEnt exhibited area under the curve (AUC) values greater than 0.8 for both training and testing datasets, indicating reliable model performance in predicting habitat suitability. The habitat suitability values for all species ranged from 0 to 1 (Figure 2).

**Figure 3:**
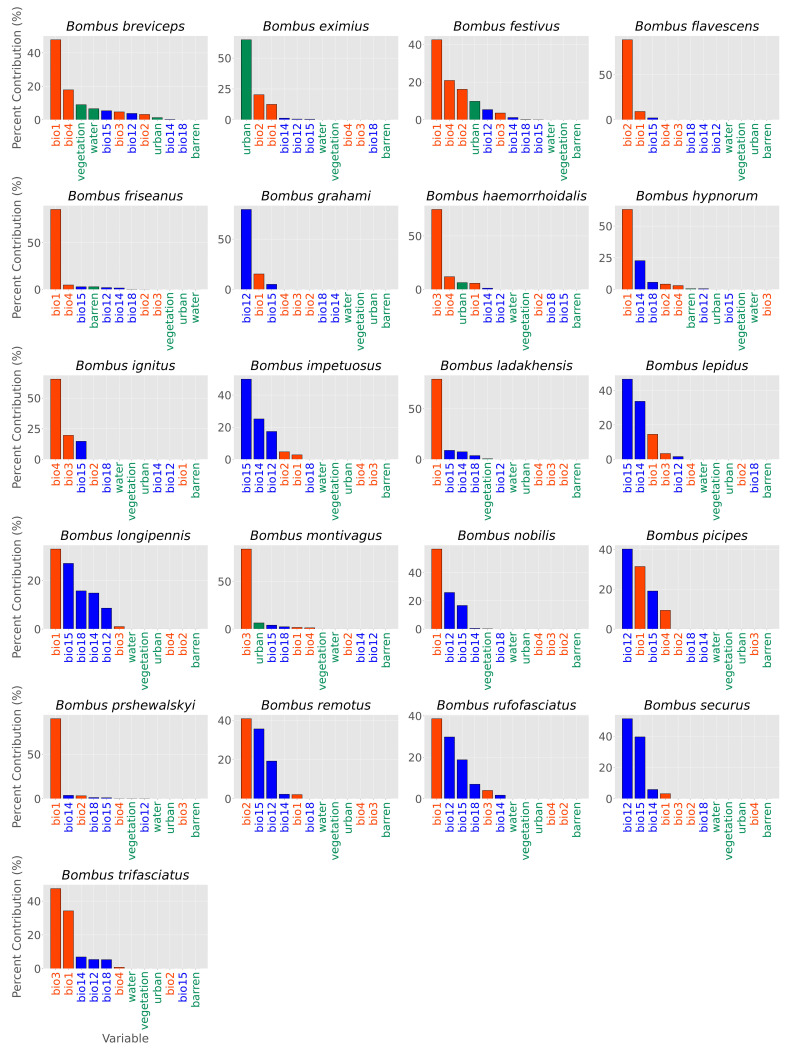
Contribution of bioclimatic and land use/land cover drivers to the spatial distribution modeling of 21 bumblebee species in Yunnan Province, China. Orange red indicates temperature-related bioclimatic drivers, blue indicates precipitation-related bioclimatic drivers, and green indicates land use/land cover drivers. bio1 = annual mean temperature; bio2 = mean diurnal range; bio3 = isothermality; bio4 = temperature seasonality; bio12 = annual precipitation; bio14 = precipitation of the driest month; bio15 = precipitation seasonality; bio18 = precipitation of the warmest quarter.

**Figure 4:**
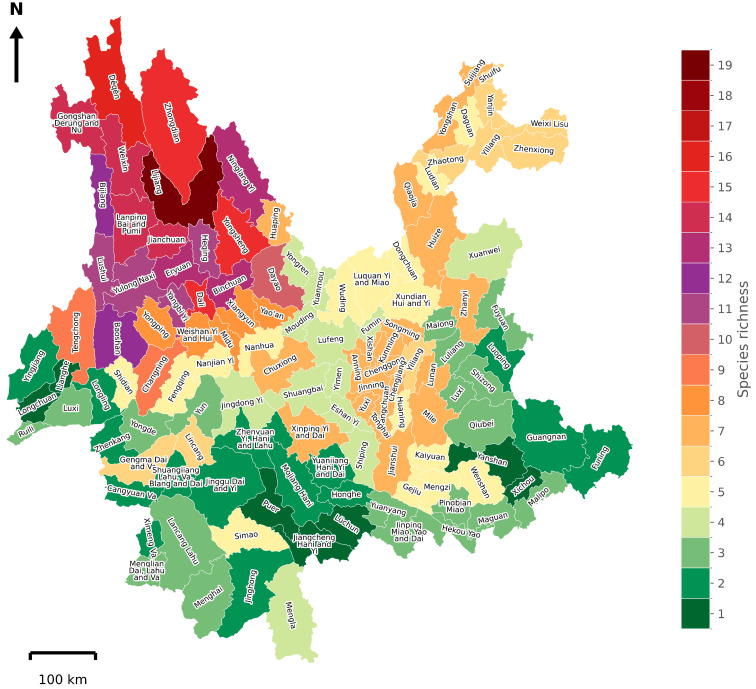
Potential species richness pattern of bumblebees per county in Yunnan Province, China.

More than 70% of species exhibited the highest habitat suitability in the northern regions of Yunnan Province. These species include *B. festivus*, *B. friseanus*, *B. grahami*, *B. hypnorum*, *B. ignitus*, *B. impetuosus*, *B. ladakhensis*, *B. lepidus*, *B. longipennis*, *B. nobilis*, *B. picipes*, *B. prshewalskyi*, *B. remotus*, *B. rufofasciatus*, and *B. securus* (Figure 2). Regarding the contribution of environmental factors to spatial distribution modeling of bumblebee species in Yunnan Province, temperature-related bioclimatic variables contributed most significantly for 15 species, whereas precipitation-related variables and LULC were most influential for remaining species (Figure 3). The highest species richness, with up to 19 species, was recorded in Lijiang County, followed by Jianchuan, Langping Bai and Pumi, and Weixin counties with 18 species each, and Zhongdian County with 16 species. All of these counties are located in the northern region of Yunnan (Figure 4).

### 3.2. Bumblebee Community Assemblage Zones

**Figure 5:**
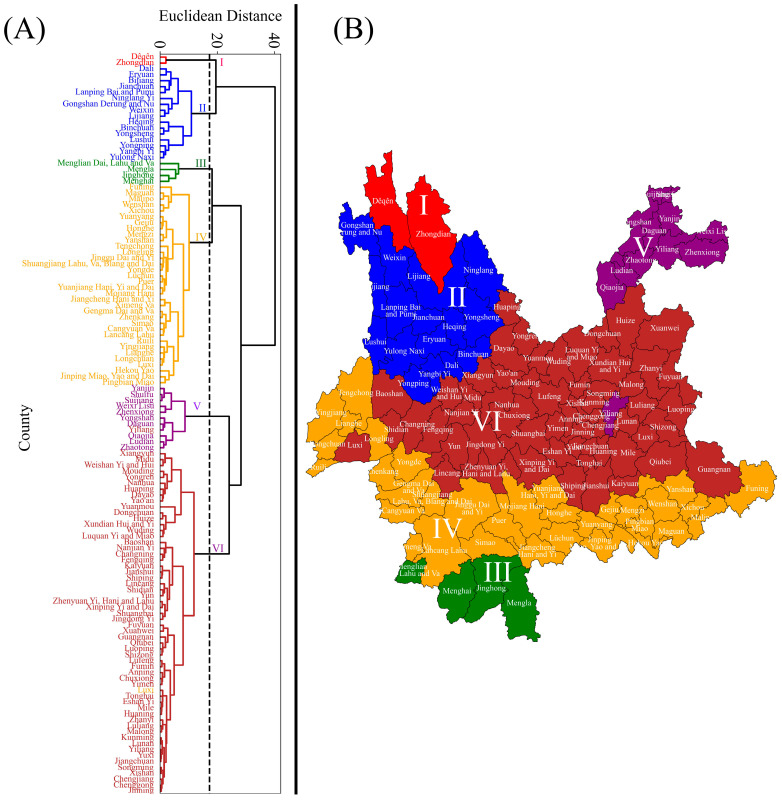
Bumblebee community assemblage zones (I, II, III, IV, V, and VI) in Yunnan Province, China, based on Ward’s agglomerative cluster analysis. Ward’s clustering, using Euclidean distance with a phenon line drawn at a threshold of 18.94, identified six distinct zones of counties (**A**). The spatial distribution of these six zones across Yunnan Province is shown in panel (**B**).

The counties of Yunnan Province were grouped into six distinct zones, as internal validation of Ward’s agglomerative cluster analysis indicated that *k* = 6 provided the most robust solution (Calinski–Harabasz = 68.6, Davies–Bouldin = 0.79, silhouette = 0.39), with cluster sizes ranging from 2 to 58 counties (Table S1; Figure 5). Most counties were assigned to Zone VI (46.40%), followed by Zone IV (27.2%), Zone II (12.8%), Zone V (8.8%), Zone III (2.4%), and Zone I (1.6%) (Figure 5A). The spatial distribution of these zones is illustrated in Figure 5B. Zone I contained the highest proportion of associated species (50%), including *B. festivus*, *B. friseanus*, *B. grahami*, *B. haemorrhoidalis*, *B. hypnorum*, *B. ladakhensis*, *B. nobilis*, *B. prshewalskyi*, *B. rufofasciatus*, and *B. securus*. Zone V comprised 30% of the species, namely *B. breviceps*, *B. convexus*, *B. eximius*, *B. flavescens*, *B. ignitus*, *B. picipes*, and *B. trifasciatus*. Zones II and IV did not contain any unique species, whereas Zone III contained only three unique species.

**Figure 6:**
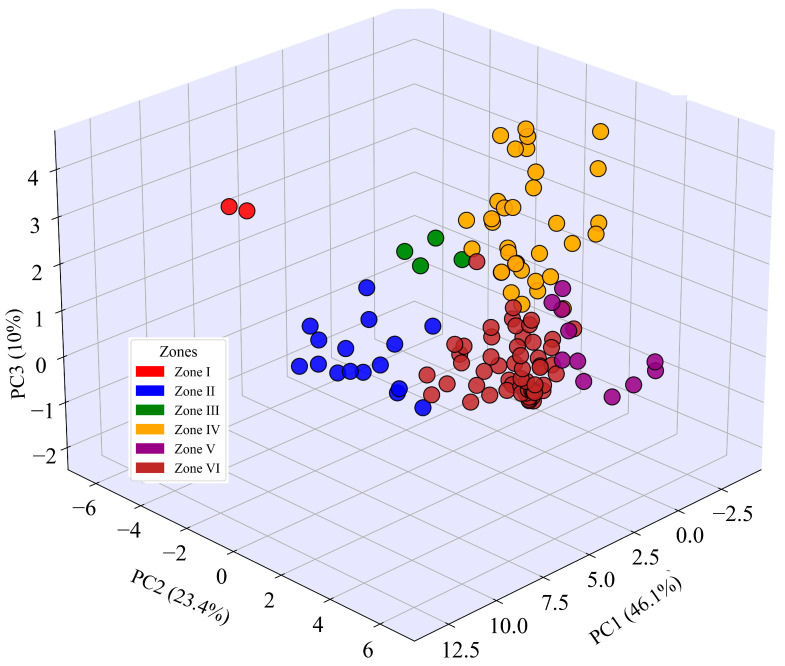
Principal component analysis (PCA) illustrating clustering patterns among counties in Yunnan Province, China, highlighting Zones I, II, III, IV, V, and VI. Each circular point represents a county within Yunnan Province.

Principal component analysis (PCA) was performed to better understand the zonation patterns of counties based on environmental variability (Figure 6). The first three PCA axes explained 79.5% of the total variability in bumblebee community composition across the 125 counties of Yunnan Province, with PCA1 explaining 46.1%, PCA2 explaining 23.4%, and PCA3 explaining 10% of the variability (Figure 6).

### 3.3. Environmental Drivers of Bumblebee Community Assemblages in Yunnan, China

**Figure 7:**
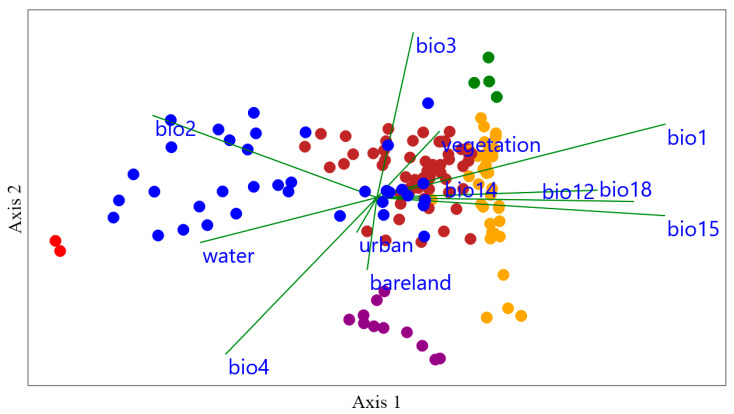
Canonical Correspondence Analysis (CCA) illustrating the relationship between bumblebee community assemblages and environmental drivers in Yunnan Province, China. Each point represents a county location, positioned according to scores on axes 1 and 2 of the CCA plot. The length and direction of arrows indicate the relative importance and influence of each environmental variable on species composition within counties. Here, bio1, bio2, bio3, and bio4 are temperature-related bioclimatic variables, whereas bio12, bio14, bio15, and bio18 are precipitation-related bioclimatic variables (www.worldclim.org), accessed on 2 January 2025. Red represents Zone I, blue represents Zone II, green represents Zone III, yellow represents Zone IV, purple represents Zone V, and maroon (brownish-red) represents Zone VI.

The assemblages of bumblebee communities in response to different environmental drivers across various zones in Yunnan Province were studied using Canonical Correspondence Analysis (CCA) (Figure 7). The variations captured by both axes 1 and 2 are indicated by their eigenvalues, with axis 1 explaining 70.65% of the variance and axis 2 explaining 14.4%. Together, these two axes significantly explain more than 84% of the total variance, supported by a permutation “*p*” value of 0.001 for both axes. This indicates that the observed community assemblage patterns (Figure 5) are significantly influenced by the 12 environmental drivers analyzed (Figure 7). In Zone I, the most influential variables were water. In Zone II, bio2 and water were identified as the primary environmental drivers. Vegetation was significantly associated with bumblebee community assemblages in Zone III. For the assemblages of bumblebees in Zone IV, bio1, bio12, bio14, bio15, and bio18 were found strongly associated. Bareland was significantly associated with bumblebee community assemblages in Zone V. Finally, in Zone VI, bio3, bio14, and vegetation were the environmental variables most strongly associated with bumblebee assemblages (Figure 7). Both axes 1 and 2 exhibited positive and negative relationships with the environmental drivers of bumblebee community assemblages (Table 1). Axis 1 had strong positive correlations with 7 out of the 12 environmental drivers, with the highest positive correlation observed with bio1 (0.87), followed by bio15 (0.82) and bio18 (0.74). Axis 2 showed positive relationships with 50% of the environmental drivers, the highest correlations being with bio3 (0.88), bio2 (0.44), and bio1 (0.39) (Table 1).

## 4. Discussion

At the local scale, it is crucial to study bumblebee species assemblages and the drivers shaping them, as this is essential for effective conservation planning [6,30]. The highest species richness was observed in the northern counties of the province (Figure 4). Previous studies have demonstrated that greater vegetation cover and natural floral resources are associated with higher species richness [31,32]. Notably, vegetation cover as a percentage of the total county area was 72% in Lijiang, 71% in Zhongdian, 62% in Weixin, and 55% in Dêqên (Figure S1). These relatively high proportions of vegetation likely contribute to the higher species richness observed in these counties (Figure 4). The higher species richness in Zones I and II of the northern region may partly reflect geographic sampling bias, as occurrence data were denser in northern Yunnan (Figure 1). Although our richness estimates were based on SDM-derived suitability predictions rather than raw records, residual bias in the input data cannot be completely excluded. Similarly, while counties with higher vegetation cover tended to support greater predicted richness, this relationship was identified descriptively and not formally tested. However, future work using standardized survey data and statistical models of richness–environment relationships would be valuable for confirming the drivers of these patterns.

In our findings, we assessed that more than 70% of species showed highest habitat suitability towards north of Yunnan and their distribution is strongly associated with the temperature-related variables (Figure 3). This finding is aligned with the previous findings where temperature was found as a most critical factor for bumblebee distribution and their activity. For example, in a recent research it was found that the temperature-related variables are strongly associated with the distribution of bumblebee species in Northern Pakistan [6]. The temperature associates have a greater role in the foraging behavior, metabolic rates, and colony dynamics [33,34,35].

The 3D PCA successfully delineated six distinct zones across the 125 counties of Yunnan (Figure 6). The three principal components together captured 79.5% of the variability in the data, highlighting the most significant underlying mechanisms or patterns of zonation [36]. For assessing patterns of biogeographic regionalization, multivariate PCA techniques have already been successfully used to study bumblebee species assemblage patterns in Gansu as well as across China [14,37]. The robust delineation of six bumblebee community assemblage zones in Yunnan indicates that the environmental drivers used in our study effectively explained the spatial structure of the community.

The spatial heterogeneity of the landscape, as well as the role of environmental gradients in shaping community structure, is well explained by Ward’s agglomerative cluster analysis [38,39] (Figure 5). The delineation of six distinct zones in Yunnan based on both PCA and Ward’s hierarchical clustering methods aligns with the approaches used in other regional studies. For example, a recent study in 2024 on Gansu Province, China, revealed four bumblebee biogeographic regions based on different environmental drivers [14]. The use of multivariate approaches for delineating bumblebee zonation patterns demonstrates the validity of our analysis, as their statistical strength in minimizing variance within clusters is supported by the fundamental principle of Ward’s original description [40].

In our analysis, we found fewer unique species in Zones III, IV, and VI, even though a larger number of counties are present in Zones IV and VI (Figure 5). The absence of a larger number of species in these zones may be due to unfavorable environmental conditions and/or insufficient sampling effort. Therefore, future surveys should include more sites in these regions to determine whether the low species richness is caused by unsuitable conditions or limited sampling [41].

Our CCA results show compelling evidence about the bumblebee community assemblage within Yunnan province based on our suitable 12 environmental drivers. Because the first two axes collectively explained over 84% variance with a significant (*p* = 0.001) permutation test, it is indicated that the variables chosen for this study are strongly influencing the community assemblages in Yunnan [42] (Figure 7). Different zones are associated with the different drivers, and especially the temperature- and precipitation-related variables were associated with the zonation of bumblebee community assemblages. This result is consistent with the previous results, where both temperature- and precipitation-related variables were found to strongly influence the distribution patterns of species [43].

A limitation of our modeling framework is that we used a single MaxEnt replicate per species, which precludes direct estimation of uncertainty (e.g., SEs or CIs) around suitability means. While our models demonstrated strong performance (AUC > 0.8), we acknowledge that future studies should incorporate replicate runs or resampling methods to quantify uncertainty in predictions, particularly for informing conservation decision-making. Further, suitability values from SDMs were used as input for CCA instead of actual abundance or occurrence data. This substitution may influence the strength of the inferred species–environment relationships. However, given the absence of standardized community-level surveys across all counties, SDM-based proxies represent a feasible and increasingly applied alternative in large-scale biogeographic studies [29]. Furthermore, our species richness maps are based on aggregated suitability outputs, rather than direct records, and may therefore be subject to pseudoreplication. These maps should be considered indicative of potential richness patterns, useful for comparative analyses but not as absolute measures of observed diversity.

Our analysis focused on climate and land use variables, and did not explicitly include biotic interactions (e.g., competition, floral resource availability), elevation, or anthropogenic pressures such as pesticide use, which may also shape bumblebee distributions in Yunnan. These factors were excluded due to limited standardized data across the province. Similarly, although Yunnan is part of the Indo-Burma biodiversity hotspot, our analysis did not compare assemblage patterns with other global hotspots. The framework developed here can be extended to future climate scenarios and to cross-hotspot comparisons, which would strengthen its conservation relevance. We also note that no formal sensitivity analysis of MaxEnt parameterization (e.g., regularization multipliers) was conducted. Future work could assess the robustness of predictions by varying modeling settings and applying model ensembles. Despite these limitations, our study provides a valuable baseline assessment of bumblebee community assemblages under current conditions.

The overall results indicated that the community assemblage of bumblebees within Yunnan is not only the product of broader climate gradients but also fine-tuned localized environmental conditions [44,45]. The clear zonation pattern observed in our study forces us to apply targeted conservation strategies which should consider both regional environmental drivers as well as local habitat characteristics of Yunnan province. Future studies should also focus on implementing more fine-scale habitat data and future climatic models to predict how these assemblages might shift in response to climate change.

## 5. Conclusions

The mapping of bumblebee community assemblages showed that both broader scale climatic gradients and fine-tuned localized conditions of habitat derive the species distribution. The patterns of distribution of 21 bumblebee species within the six zones of Yunnan province indicated that a particular zone is associated with species. For example, Zone I was identified as a hotspot region for bumblebee species in Yunnan Province due to its rich vegetation cover and abundant floral resources. In contrast, Zones IV and VI, despite their larger areas, showed lower species representation, suggesting the need for further targeted sampling.

## Figures and Tables

**Table 1:** Relationship of environmental drivers with axes 1 and 2 of Canonical Correspondence Analysis (CCA).

Environmental Drivers	Axis 1 of CCA Plot	Axis 2 of CCA Plot
bareland	−0.03	−0.38
bio1	0.83	0.39
bio2	−0.64	0.44
bio3	0.10	0.88
bio4	−0.43	−0.83
bio12	0.63	0.04
bio14	0.19	0.11
bio15	0.82	−0.10
bio18	0.74	−0.02
urban	−0.06	−0.18
vegetation	0.18	0.35
water	−0.51	−0.24

## Data Availability

All the data generated or analyzed during this study are included in this published article.

## Supplementary Materials

The following supporting information can be downloaded at: https://www.mdpi.com/article/10.3390/biology14091222/s1. Table S1. Validation of hierarchical clustering on county × suitability dataset (k = 2–10). Higher Silhouette and Calinski–Harabasz values and lower Davies–Bouldin indicate better clustering. Chosen k = 6. Figure S1. Land use Land cover classification of Yunnan, China.
