# Supplementary material for: Mapping Bumblebee Community Assemblages and Their Associated Drivers in Yunnan, China
Source: Biology (Basel). 2025 Sep 9;14(9):1222. doi: 10.3390/biology14091222 (PMC12467643; doi:10.3390/biology14091222)
Supplement: Supplementary file 1 [file biology-14-01222-s001.zip › biology-3841885-supplementary.pdf]

biology

MDPI

Table S1. Validation of hierarchical clustering on county × suitability dataset (k = 2–10). Higher Silhouette and Calinski–Harabasz values and lower Davies–Bouldin indicate better clustering. Chosen k = 6.

k	Silhouette (↑)	Calinski–Harabasz (↑)	Davies–Bouldin (↓)
2	0.46	58.92	0.96
3	0.32	57.21	1.16
4	0.36	60.64	0.99
5	0.38	62.82	0.85
**6**	**0.39**	**68.64**	**0.79**
7	0.33	66.01	0.90
8	0.33	64.40	0.97
9	0.33	63.61	0.97
10	0.33	62.12	0.94

 https://doi.org/10.3390/xxxxx

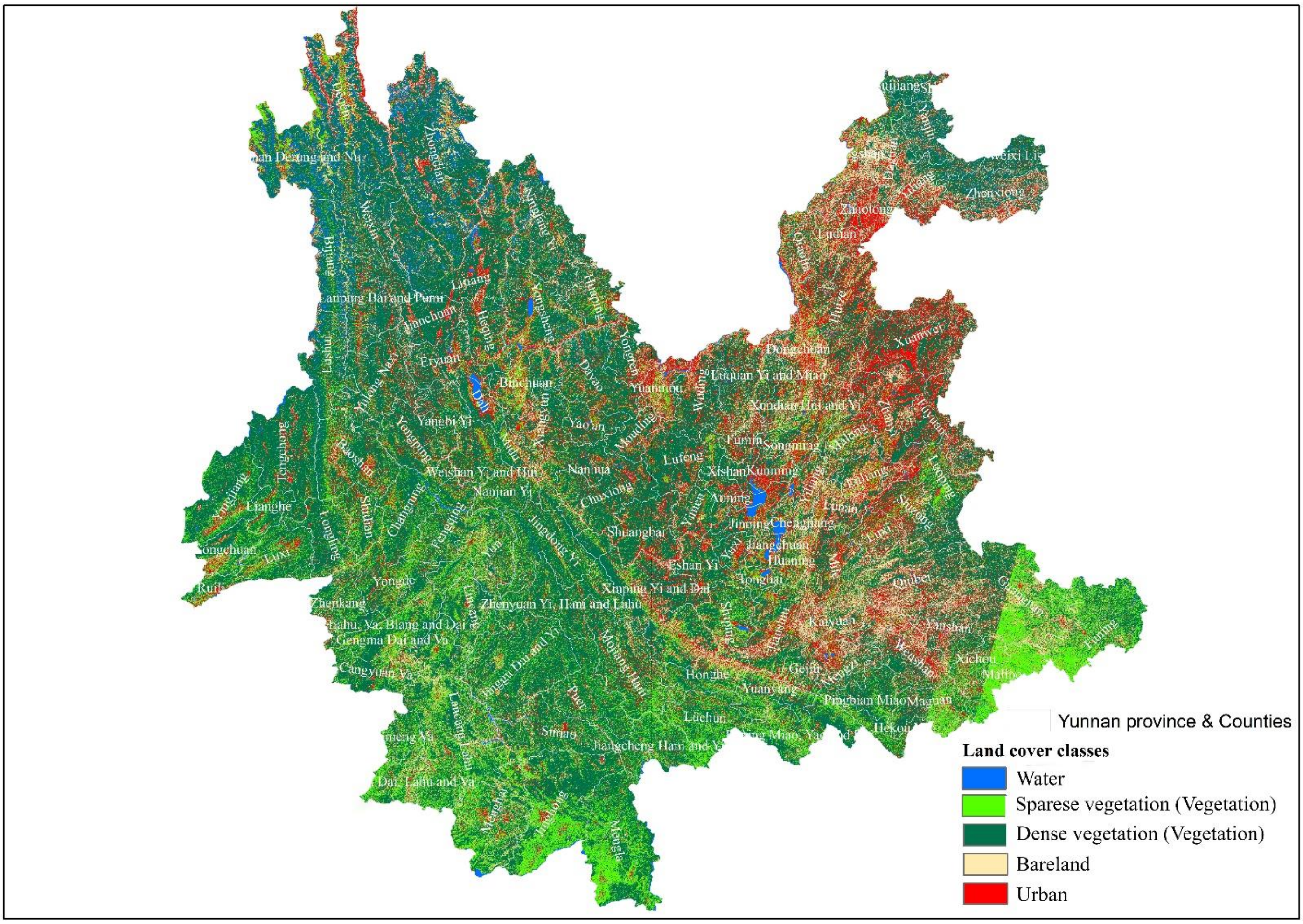

Figure S1. Land use Land cover classification of Yunnan, China.

https://doi.org/10.3390/xxxxx
